# Maternal Obesity Modulates Cord Blood Concentrations of Proprotein Convertase Subtilisin/Kexin-type 9 Levels

**DOI:** 10.1210/jendso/bvae031

**Published:** 2024-02-20

**Authors:** Dimitrios Rallis, Aimilia Eirini Papathanasiou, Helen Christou

**Affiliations:** Brigham and Women's Hospital, Harvard Medical School, Boston, MA 02115, USA; Neonatal Intensive Care Unit, University of Ioannina, Faculty of Medicine, Ioannina 45110, Greece; Brigham and Women's Hospital, Harvard Medical School, Boston, MA 02115, USA; Brigham and Women's Hospital, Harvard Medical School, Boston, MA 02115, USA

**Keywords:** body mass index, cardiovascular disease, gestational diabetes, hypercholesterolemia, metabolic syndrome

## Abstract

**Context:**

In utero exposure to maternal obesity or diabetes is considered a pro-inflammatory state.

**Objective:**

To evaluate whether cord blood proprotein convertase subtilisin/kexin-type 9 (PCSK9), which is regulated by inflammation and metabolic derangements, is elevated in neonates born to overweight, obese, or diabetic mothers.

**Methods:**

A retrospective study in full-term neonates born between 2010 and 2023, at Brigham and Women's Hospital. There were 116 neonates included in our study, of which 74 (64%) were born to overweight/obese mothers and 42 (36%) were born to nonoverweight/nonobese mothers.

**Results:**

Neonates born to overweight/obese mothers had significantly higher cord blood concentrations of PCSK9 compared with neonates born to nonoverweight/nonobese group (323 [253-442] ng/mL compared with 270 [244-382] ng/mL, *P* = .041). We found no significant difference in cord blood concentrations of PCSK9 between neonates of diabetic mothers compared with neonates of nondiabetic mothers. In multivariate linear regression analysis, higher cord plasma PCSK9 concentration was significantly associated with maternal overweight/obesity status (b = 50.12; 95% CI, 4.02-96.22; *P* = .033), after adjusting for gestational age, birth weight, male sex, and intrauterine growth restriction.

**Conclusion:**

Neonates born to mothers with overweight/obesity have higher cord blood PCSK9 concentrations compared with the nonoverweight/nonobese group, and higher cord blood PCSK9 concentrations were significantly associated with maternal overweight/obesity status, after adjusting for perinatal factors. Larger longitudinal studies are needed to examine the role of PCSK9 in the development of metabolic syndrome in high-risk neonates born to overweight, obese, or diabetic mothers.

In utero exposure to maternal obesity or diabetes is associated with a pro-inflammatory state [1, 2] and increased risk of cardiometabolic diseases in the offspring [3], including obesity, elevated blood pressure, impaired insulin/glucose homeostasis, and abnormal lipid profiles [4-6]. Maternal metabolic changes during pregnancy lead to perturbations in fetal insulin sensitivity, lipid metabolism, and placental energy metabolism [3]. The placenta produces an array of signaling molecules that control crosstalk between the mother and the fetus and regulate the fetal metabolism including lipoprotein homeostasis [7]; however, the mechanisms regulating fetal lipoprotein homeostasis in high-risk pregnancies have not been fully evaluated.

Proprotein convertase subtilisin/kexin type 9 (PCSK9) is primarily derived from hepatocytes; it is also expressed in other tissues such as small intestine, kidney, pancreas, and immune system [8] and binds low-density lipoprotein (LDL) receptors thereby leading to higher serum LDL cholesterol concentrations [9, 10]. The large molecular weight of PCSK9 likely precludes its transplacental transfer, but PCSK9 is detectable in cord blood and may regulate fetal lipid levels [10, 11]. Previous studies showed that PCSK9 concentrations in cord blood or the serum of high-risk neonates (ie, neonates born premature, with intrauterine growth restriction, or small or large for gestational age [GA]) differ significantly compared with neonates born at term, without growth restriction, or with appropriate birth weight for GA [12-14]. However, the impact of maternal overweight/obesity and/or diabetes on the concentrations of PCSK9 in the neonatal-placental circulation is unknown. In the current study, we examined the cord blood concentrations of PCSK9 in neonates born to overweight/obese compared with neonates born to nonoverweight/nonobese and diabetic compared with nondiabetic mothers.

## Materials and Methods

We conducted a retrospective case-control study at Brigham and Women's Hospital, Boston, MA, USA. The study was approved by the Mass General Brigham Human Research Committee, which is the institutional review board of Mass General Brigham, on behalf of the Brigham and Women's Hospital (Protocol Number 2012P000384/02.10.2012). The Human Research Committee waived the requirement to obtain a consent. From our biorepository of cord blood samples from 2013 to 2023, we included 116 available samples from neonates born at ≥37 weeks of GA. Prepregnancy maternal body mass index (BMI) was calculated based on the maternal weight and height that were measured at the time of the initial pregnancy assessment, according to the formula BMI = weight/height^2^. Maternal BMI of 18.5 to 24.9 was classified as normal, BMI of 25 to 29.9 as overweight, and BMI ≥30 as obesity per accepted standards [15]. Maternal diabetes was diagnosed according to the guidelines of the American Diabetes Association, including preexisting diabetes and gestational diabetes mellitus [16]. We compared cord blood PCSK9 concentrations in neonates born to overweight/obese vs nonoverweight/nonobese mothers and to diabetic vs nondiabetic mothers.

We collected the perinatal data for neonates including GA, birth weight, length, and head circumference, sex, intrauterine growth restriction, delayed cord clamping, parity, maternal age, and race, mode of conception and delivery, multiple pregnancy, gestational hypertension, prolonged rupture of membranes, chorioamnionitis, Apgar score in the first and fifth minutes, resuscitation at birth, and survival.

### Measurement of Cord Blood PCSK9 Concentration

Mixed arteriovenous blood samples were collected at the time of delivery from the doubly clamped umbilical cord, in EDTA tubes, and were immediately centrifuged. The supernatant plasma was kept frozen at −80 °C until assay.

Plasma PCSK9 levels were determined in a single batch in duplicate using a sandwich enzyme-linked immunosorbent assay kit (Bio Vendor Laboratory Medicine, catalog number RD191473200R, RRID AB_3083527; limit of detection: 9 pg/mL). The intra- and interassay coefficients of variation were 5.2% to 5.3% and 4.0% to 7.5%, respectively.

### Statistical Analysis

Continuous variables were expressed as mean ± standard deviation or median (interquartile range), as appropriate. The normality of the distributions of continuous variables was assessed by the Kolmogorov-Smirnov test. Comparisons of continuous variables between 2 groups were performed using the Student unpaired *t*-test or the nonparametric Mann-Whitney test. Comparison of cord blood PCSK9 levels between more than2 groups was performed using the nonparametric Kruskal-Wallis test. Categorical variables were expressed as n (percentage %) and compared with the χ^2^ test or Fisher exact test. The correlation between prepregnancy maternal BMI with the cord blood PCSK9 concentration in neonates born to overweight/obese, nonoverweight/nonobese, diabetic, and nondiabetic mothers was examined with Spearman rho. A multivariate linear regression model with generalized estimating equations to account for multiples in our patient cohort was used to examine the association of PCSK9 levels (dependent variable) with maternal overweight/obesity, adjusted for GA, birth weight, sex, and intrauterine growth restriction (independent variables). Coefficients and 95% CIs were calculated.

All tests were 2-sided and a *P* < .05 was considered statistically significant (alpha .05). A power analysis revealed that a sample size of 116 neonates is sufficient to detect a difference of 15% in cord plasma PCSK9 concentration between groups, with a power of 0.8 and a type I error of 0.05. The data were analyzed using SPSS Statistics (IBM SPSS Statistics for Windows, Version 24.0. Armonk, NY, USA).

## Results

**Figure 1.**
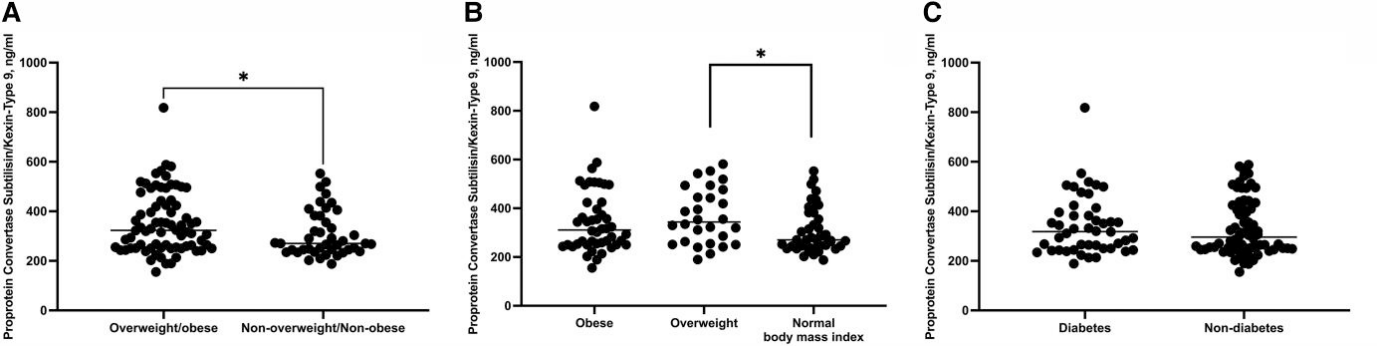
(A) Proprotein convertase subtilisin/kexin type 9 (PCSK9) in neonates born to overweight/obese mothers compared with neonates born to mothers with normal body mass index. (B) PCSK9 in neonates born to mothers with normal body mass index, overweight, and obesity. (C) PCSK9 in neonates born to diabetic mothers compared with neonates born to nondiabetic mothers.

Among the 116 neonates included in our study, 74 (64%) were born to overweight/obese mothers and 42 (36%) were born to nonoverweight/nonobese mothers. There were no differences in perinatal characteristics between the 2 groups, except for a significantly higher birthweight in favor of the overweight/obese group (Table 1). The mean GA in the overweight/obese group was 38.5 ± 0.8 weeks and the mean birth weight was 3551 ± 585 g, compared with a mean GA of 38.2 ± 0.8 (*P* = .083) and birth weight of 3225 ± 514 g (*P* = .003) in the nonoverweight/nonobese group (Table 1). Neonates in the overweight/obese group had significantly higher cord blood concentrations of PCSK9 compared with neonates in the nonoverweight/nonobese group (323 [253-442] ng/mL compared with 270 [244-382] ng/mL, *P* = .041) (Fig. 1A). When analyzed by maternal prepregnancy BMI, a significant difference was found between cord blood PCSK9 concentrations in neonates of overweight mothers vs neonates born to mothers with normal prepregnancy BMI (343 [259-460] ng/mL vs 270 [244-382] ng/mL, *P* = .023) (Fig. 1B).

**Table 1.** Perinatal characteristics of neonates born to overweight or obese mothers and neonates born to mothers with normal BMI

	Overweight/obese (n = 74)	Normal BMI (n = 42)	*P*
PCSK9, ng/mL	323 (253-442)	270 (244-382)	.041
Gestational age, weeks	38.5 ± 0.8	38.2 ± 0.8	.083
Birthweight, g	3551 ± 585	3225 ± 514	.003
Birth length, cm	49.8 ± 2.5	49.4 ± 2.6	.440
Head circumference, cm	35.3 ± 2.6	34.4 ± 1.6	.166
Sex, male	39 (53%)	23 (55%)	.849
Intrauterine growth restriction	6 (8%)	5 (12%)	.524
Maternal diabetes	32 (43%)	14 (33%)	.328
Maternal BMI	34.1 ± 4.8	24.1 ± 3.1	<.001
Maternal BMI category			<.001
Normal (18.5-24.9) Overweight (25-29.9) Obese (≥30)	- 28 (38%) 46 (62%)	42 (100%) - -	
Delayed cord clamping	54 (73%)	33 (79%)	.656
First parity	24 (33%)	13 (31%)	.289
Maternal age, years	34.1 ± 4.8	34.7 ± 4.6	.520
Maternal race			.286
White/Caucasian	41 (55%)	29 (69%)	
Black/African American Hispanic Asian Other	14 (19%) 10 (14%) 8 (11%) 1 (1%)	3 (7%) 4 (10%) 4 (10%) 2 (4%)	
Conception, in vitro fertilization	4 (5%)	6 (14%)	.165
Delivery mode, cesarean section	73 (99%)	41 (98%)	1.000
Twin pregnancy	1 (1%)	2 (5%)	.297
Gestational hypertension	15 (20%)	4 (10%)	.192
Prolonged rupture of membranes	3 (4%)	1 (2%)	1.000
Chorioamnionitis	1 (1%)	2 (4%)	.297
Apgar first minute	8 (8-8)	8 (8-8)	.141
Apgar fifth minute	9 (9-9)	9 (9-9)	.319
Resuscitation	8 (11%)	3 (7%)	.744
Survival	74 (100%)	42 (100%)	1.000

Continuous variables are expressed as mean ± SD or median (interquartile range). *P* values of Student *t*-test or Mann-Whitney test. Categorical variables are expressed as n (%). *P* values of χ^2^ test or Fisher exact test.

Abbreviations: BMI, body mass index; PCSK9, proprotein convertase subtilisin/kexin type 9.

Of the 116 neonates, 46 (40%) were born to diabetic mothers and 70 (60%) were born to nondiabetic mothers. There were no differences in perinatal characteristics between neonates born to diabetic mothers and neonates born to nondiabetic mothers except for a significantly higher birth weight among infants of diabetic mothers (3567 ± 687 g vs 3345 ± 482 g; *P* = .043) (Table 2). We found no significant difference in cord blood concentrations of PCSK9 between neonates of diabetic mothers compared with neonates of nondiabetic mothers (318 [250-395] ng/mL compared with 296 [250-418] ng/mL, *P* = .729) (Fig. 1C). Also, among the 74 neonates of overweight/obese mothers, we found no significant difference in cord blood concentrations of PCSK9 between the 32 (43%) neonates of diabetic mothers compared with the 42 (57%) neonates of nondiabetic mothers (314 [247-379] ng/mL compared with 340 [255-493] ng/mL, *P* = .230).

**Table 2.** Perinatal characteristics of neonates born to diabetic mothers and neonates born to non-diabetic mothers

	Diabetes (n = 46)	Nondiabetes (n = 70)	*P*
PCSK9, ng/mL	318 (250-395)	296 (250-418)	.729
Gestational age, weeks	38.2 ± 0.9	38.5 ± 0.8	.154
Birthweight, g	3567 ± 687	3345 ± 482	.043
Birth length, cm	49.8 ± 2.8	49.6 ± 2.3	.658
Head circumference, cm	35.7 ± 3.3	34.6 ± 1.5	.066
Sex, male	26 (57%)	36 (51%)	.704
Intrauterine growth restriction	5 (11%)	6 (9%)	.751
Maternal BMI	31.7 ± 7.2	30.1 ± 6.9	.253
Maternal BMI category			.188
Normal (18.5-24.9) Overweight (25-29.9) Obese (≥30)	14 (30%) 9 (20%) 23 (50%)	28 (40%) 19 (27%) 23 (33%)	
Delayed cord clamping	35 (76%)	52 (74%)	1.000
First parity	19 (41%)	18 (26%)	.120
Maternal age	35.0 ± 4.6	34.0 ± 4.8	.276
Maternal race			.556
White/Caucasian	29 (63%)	41 (59%)	
Black/African American Hispanic Asian Other	5 (11%) 7 (15%) 5 (11%) -	12 (17%) 7 (10%) 7 (10%) 3 (4%)	
Conception, in vitro fertilization	4 (9%)	6 (9%)	1.000
Delivery mode, cesarean section	44 (96%)	70 (100%)	.155
Twin pregnancy	3 (7%)	—	.060
Gestational hypertension	6 (13%)	13 (19%)	.458
Prolonged rupture of membranes	1 (2%)	3 (4%)	1.000
Chorioamnionitis	1 (2%)	2 (3%)	1.000
Apgar first minute	8 (8-8)	8 (8-8)	.570
Apgar fifth minute	9 (9-9)	9 (9-9)	.239
Resuscitation	7 (15%)	4 (6%)	.110
Survival	46 (100%)	70 (100%)	1.000

Continuous variables are expressed as mean ± SD or median (interquartile range). *P* values of Student's *t*-test or Mann-Whitney test. Categorical variables are expressed as n (%). *P* values of χ^2^ test or Fisher exact test.

Abbreviations: BMI, body mass index; PCSK9, proprotein convertase subtilisin/kexin type 9.

Prepregnancy maternal BMI was significantly associated with cord blood PCSK9 only in neonates born to nonoverweight/nonobese mothers (*r* = 0.495, *P* = .002). This association was not significant in any other neonatal-maternal group (Table 3).

**Table 3.** Spearman rho analysis of the association of prepregnancy maternal body mass index with cord blood PCSK9

	rho	*P*
Overweight/obesity	0.142	.231
Non-overweight/non-obesity	0.495	.002
Diabetes	0.043	.783
Non-diabetes	0.180	.146

Abbreviation: PCSK9, proprotein convertase subtilisin/kexin type 9.

In multivariate linear regression analysis, higher cord plasma PCSK9 concentration was significantly associated with maternal overweight/obesity status (b = 50.12; 95% CI, 4.02-96.22; *P* = .033) after adjusting for GA (b = −16.60; 95% CI, −96.22 to −10.02; *P* = .219), birth weight (b = −0.01; 95% CI, −0.05 to −0.04; *P* = .963), male sex (b = −8.83; 95% CI, −52.86 to −35.19; *P* = .692), and intrauterine growth restriction (b = 13.45; 95% CI, −70.32 to −97.24; *P* = .751) (Table 4).

**Table 4.** Multivariate linear regression (generalized estimating equations) analysis of the PCSK9 (dependent variable) with variables that were significant in univariate analysis, namely obesity/overweight, gestational age, birth weight, sex, and intrauterine growth restriction (independent variables)

	b	95% CI	*P*
PCSK9			
Overweight/obesity	50.12	4.02-96.22	.033
Gestational age	−16.60	(−96.22)-10.02	.219
Birth weight	−0.01	(−0.05)-0.04	.963
Sex, male	−8.83	(−52.86)-35.19	.692
Intrauterine growth restriction	13.45	(−70.32)-97.24	.751

Abbreviation: PCSK9, proprotein convertase subtilisin/kexin type 9.

## Discussion

Our study demonstrates that cord blood PCSK9 concentrations are significantly higher in neonates born to overweight/obese mothers compared with neonates born to nonoverweight/nonobese mothers. Prepregnancy maternal BMI is significantly associated with cord blood PCSK9 in neonates born to nonoverweight/nonobese mothers, whereas higher cord blood PCSK9 concentrations are significantly associated with maternal overweight/obesity status, after adjusting for GA, birth weight, sex, and intrauterine growth restriction.

PCSK9 regulates circulating LDL cholesterol levels by promoting the degradation of LDL receptors [9]. It is mainly produced in the liver but also expressed in various cells throughout the body, including small intestinal enterocytes, vascular endothelial and smooth muscle cells [17], cardiomyocytes [18], macrophages [19], and various cells in the placenta [20]. In fetal life, PCSK9 expression has been first detected in the liver at embryonic day 9 and in the skin, kidney, small intestine, and cerebellum at embryonic day 15 [21]. In tissue culture and animal studies, PCSK9 expression is increased in response to hypoxia, cardiomyocyte injury [22], systemic inflammation [23], hemodynamic shear stress, and oxidative stress [24-26]. In human studies in adults, serum PCSK9 levels are positively associated with acute myocardial infarction [27] and high-sensitivity C-reactive protein levels [28], whereas PCSK9 inhibitors such as evolocumab or alirocumab reduce the risk of recurrent ischemic cardiovascular events [29, 30]. In pregnant women, serum PCSK9 concentrations at delivery are significantly higher compared with nonpregnant women, and PCSK9 concentrations in cord blood are significantly lower than the corresponding maternal concentrations, indicating the unlikely contribution of maternal PCSK9 to cord blood concentrations [10]. Moreover, PCSK9 was previously associated with urgent cesarean delivery [31]. Previous studies in neonates showed that PCSK9 concentrations were significantly higher in neonates born small or large for GA compared with neonates born appropriate for GA. Also, PCSK9 concentrations in neonates showed a significant correlation with total and LDL cholesterol [12]. Another study in newborns showed that neonatal PCSK9 concentrations were a significant predictor of fetal LDL-cholesterol levels independent of small-for-GA status in multivariate analysis [13]. Our findings, demonstrating that cord blood PCSK9 concentrations were significantly higher in neonates born to overweight/obese mothers compared with neonates born to mothers with normal prepregnancy BMI, lend support to the notion that the proinflammatory state of being overweight, or obese, leads to elevated PCSK9 [32].

Maternal overweight status, obesity, and diabetes during pregnancy are considered proinflammatory states [1, 2]. Obesity is known to cause chronic endothelial activation and dysfunction [33, 34], excessive production of reactive oxygen species [35, 36], hyperinsulinemia and insulin resistance [37], and dyslipidemia [1]. In addition, exposure to an obesogenic maternal environment has been associated with abnormalities in placental metabolic regulation, leading to epigenetic modifications [38-40]. Placentas from diabetic or obese pregnancies show decreased expression of genes implicated in cholesterol utilization and LDL receptors, independent of the level of maternal cholesterol but likely resulting from high fetal insulin levels [19, 40]. Epigenetic remodeling during early life could constitute a molecular mechanism through which intrauterine stimuli can have an impact on gene regulation and DNA damage and repair [41-44]. A recent study showed hypomethylation of the PCSK9 gene in placentas of diabetic pregnancies compared with controls [45], and DNA methylation alterations in blood cells of offspring born to obese mothers have also been previously demonstrated [6].

Higher circulating PCSK9 levels were found in obese compared with nonobese adult subjects [46, 47], and female obese subjects with type 2 diabetes had the highest levels of PCSK9 within a group of young adults aged 15 to 26 years [9]. Taken together, these previous studies support that female sex and proinflammatory states such as overweight/obesity status, as well as intrauterine growth restriction and prematurity are associated with higher circulating PSCK9 concentrations, suggesting a possible impact of estrogens, epigenetics, and gestational maturation on metabolic states [12-14]. In our study, cord blood PCSK9 concentration was significantly associated with maternal overweight/obesity status, after adjusting for GA, birth weight, sex, and intrauterine growth restriction. We conclude that PCSK9 may be a useful biomarker of lipoprotein metabolism in fetal life because PCSK9 levels in the neonatal-placental circulation are susceptible to several conditions that affect lipoprotein regulation including maternal overweight status/obesity.

Our study has several limitations. Given the known association between obesity and insulin resistance, it is possible that we included women with undiagnosed insulin resistance within our nondiabetic mother group, two thirds of which were overweight or obese. In addition, we do not have data on maternal lipid profiles, circulating PCSK9 concentrations, or markers of glucose metabolism. Although in previous studies, PCSK9 levels have been correlated with markers of blood glucose homeostasis in patients with type 2 diabetes [48], in our retrospective study, we used stored samples that were of limited quantity and were not appropriately processed for lipid analysis, inflammation markers, or markers of glucose metabolism. Future longitudinal studies would be important in further delineating the relationship between maternal obesity or diabetes and infant PCSK9 levels.

## Conclusions

Higher cord blood PCSK9 concentrations are significantly associated with maternal overweight/obesity status, after adjustment for GA, birth weight, sex, and intrauterine growth restriction; this finding may warrant larger, prospective, longitudinal studies to examine the utility of PCSK9 as a biomarker of lipoprotein metabolic profile in high-risk neonates and its potential predictive role in later metabolic syndrome.

## Data Availability

Restrictions apply to the availability of some, or all data generated or analyzed during this study to preserve patient confidentiality or because they were used under license. The corresponding author will on request detail the restrictions and any conditions under which access to some data may be provided.

## Abbreviations

BMI: body mass index
GA: gestational age
LDL: low-density lipoprotein
PCSK9: proprotein convertase subtilisin/kexin-type 9
